# Identification of novel epitopes from human papillomavirus type 18 E7 that can sensitize PBMCs of multiple HLA class I against human cervical cancer

**DOI:** 10.1186/s12967-014-0229-7

**Published:** 2014-08-21

**Authors:** Sunghoon Kim, Hye Won Chung, Kyoung-Ryul Lee, Jong-Baeck Lim

**Affiliations:** Department of Gynecology and Oncology, Yonsei University College of Medicine, Seoul, Republic of Korea; Division of Gastroenterology, Department of Internal Medicine, International ST. Mary’s Hospital, Incheon, Republic of Korea; Department of Clinical Research, Seoul Medical Science Institute, Seoul, Republic of Korea; Department of Laboratory Medicine, Yonsei University College of Medicine, Seoul, 135-720 Republic of Korea

**Keywords:** HLA class I, HPV18 E7, Immunotherapy, Cervical cancer, Epitopes

## Abstract

**Background:**

To identify the novel epitopes from the human papillomavirus type 18 E7 which can sensitize PBMCs of four different major HLA class I A allele.

**Methods:**

Twenty-four synthetic overlapping 15-amino acid peptides were screened by measuring the frequency of CD8^+^ cytotoxic T lymphocytes (CTLs)-producing interferon-γ (IFN-γ) by using flow cytometry and ELISpot assays and selected peptides were validated for cytolytic activity by using the ^51^Cr release assay. Truncated peptides in the selected epitopes were tested to determine the important residues using ELISpot and ^51^Cr release assay.

**Results:**

Among 24 peptides, E7_81-95_DDLRAFQQLFLNTLS (#21) and E7_89-103_LFLNTLSFVCPWCAS (#23) induced significantly higher Th 1 response including IFN-γ production and *in vitro* cytotoxicity of PBMCs of four different HLA-A alleles against cervical cancer cells than that of other peptides and the negative control (no peptide sensitization). In E7_81–95_ (#21), amino acid position 81, 82 (N-terminus) and 92, 94, 95 (C-terminus) for HLA-A*02:02 and 24:02, and 81, 82 (N-terminus) and 92, 95 (C-terminus) for HLA-A*11:01 and 33:03 were important to elicit Th1 response of PBMCS. In E7_89–103_ (#23), residue 100 and103 (C-terminus) were important to elicit the CD8^+^ CTL response in HLA-A*02:01, 11:01 and 33:03 and 100, 101, and 103 (C-terminus) were important to elicit the CD8^+^ CTL response in HLA-A*24:02.

**Conclusions:**

E7_81–95_ (#21) and E7_89–103_ (#23) were identified as novel epitopes from HPV18 E7 which could sensitized PBMCs of four different HLA class I (HLA-A*02:01, 24:02, 11:01 and 33:03). These epitopes could be useful for immune monitoring and immunotherapy for HPV 18+ cervical cancer.

## Introduction

A number of previous studies have reported that various types of cancer are caused by Infectious pathogens such as human papilloma virus (HPV), *Helicobacter pylori*, and hepatitis viruses [1,2]. HPV has been proven as a major causative pathogen of cervical cancer, anal cancer and nasopharyngeal cancer. Cervical cancer is the third most commonly diagnosed cancer and second leading cause of cancer-related death worldwide in women [3,4] and the incidence of nasopharyngeal cancer have been markedly increased during last decade in both men and women [5–8]. Therefore, the control of HPV infection is critical for the prevention or treatment of not only in precancerous states, but also in HPV-induced cancers. Among over 100 subtypes of HPV, HPV 16 and 18 are most important subtypes and about 70% of cervical cancers are associated with these two types of HPV [9].

Although currently available two prophylactic HPV vaccines have been shown to effectively prevent HPV-associated anogenital disease in young women and men [8,9], only 32% of teenagers who qualify for immunization have received all three recommended doses of the vaccine in the USA [10]. These vaccines did not show a therapeutic effect on pre-existing cervical infection or cervical lesions [11,12]. Moreover, conventional primary treatments for early stage cervical cancer showed a recurrence rate of about 15% [13,14]. In the case of late stage or recurrent disease, treatment results are relatively poor. Thus, more effective ways which can control or treat HPV infection and HPV-related cancer should be needed.

Spontaneous regression of HPV-infected precancerous lesions has been reported in patients who had strong HPV-specific Th1-biased T-cell responses [15–18]. In particularly, a strong CD8+ cytotoxic T lymphocyte (CTL) response could play an important role not only for control of HPV infection, but also for treatment of HPV-induced cervical cancer. The peptides from the HPV protein could successfully sensitize and expand HPV-specific CTLs and these peptides can be used for immune monitoring as well as for vaccine or immune therapy against HPV-induced disease [19,20]. However, one of the major drawback limiting the use of peptide-based immune monitoring or immunotherapy is that identification of at least one immune dominant epitopes from HPV molecules for each HLA class I alleles are needed for wide clinical application because CD8+ CTLs from patients or donors who have different HLA class I genetic background recognized different epitopes of HPV molecules. For overcome this drawback of peptide-based immune monitoring or immunotherapy, the identification of single peptides that can bind to multiple HLA types have been investigated and theses could lead to effective coverage of the human population by a peptide-based immune monitoring or immunotherapy. However, very few studies have addressed PBMC recognition of single peptides in a genetically heterogeneous group previously exposed to an infectious agent or cancer [21–24].

In this study, we report that novel single peptides that can bind to 4 different HLA class I which are A*02:01, A*24:02, A*11:01 and A*33:03, most frequent allele in human race. Theses single peptides were successfully elicit HPV 18-speciifc Th 1 responses of PBMCs.

## Methods

### Donors and peripheral blood mononuclear cells

Peripheral blood mononuclear cells (PBMCs) were collected from four HLA class I (HLA-A*02:01, A*24:02, A*11:01, A*33:03) healthy donors. Human leukocyte antigen (HLA) class I genotypes were determined by the HLA laboratory at the Seoul Medical Science Institute (Seoul, South Korea) via sequence-specific polymerase chain reaction using genomic DNA. PBMCs were isolated via density gradient centrifugation using Ficoll-Hypaque 1.077 (Pharmacia Biotech, Wilkstrom, Sweden). The mononuclear cells were cryopreserved at −160°C in human AB + serum containing 10% dimethylsulfoxide (DMSO; Sigma, St Louis, MO, USA). This research was approved by the institutional review board of Yonsei University Health System, and all participants provided written informed consent.

### Synthesis of HPV16 E7 peptides

A total of twenty-four, 15-amino acid peptides spanning the HPV18 E7 protein that overlapped by 11 residues, were synthesized commercially (purity of >95%; A & Pep, Yeongi-gun, South Korea; Table 1). After selection of the immune dominant candidate 15-amino acid peptides which were restricted to each HLA class I from the peptide library by screening and confirmation tests, truncated peptides spanning the candidate 15-amino acid peptides were synthesized (Table 2). The peptides were diluted to working solution concentrations (1 μg/μL) in diethylpyrocarbonate-treated water (Invitrogen, Carlsbad, CA, USA) containing 1% DMSO and stored at −80°C before testing.

**Table 1:** **15-amino acid overlapping peptides spanning the HPV type 18 E7 protein**

**Number**	**Amino acid position**	**Amino acid sequence**
1	1-15	MHGPKATLQDIVLHL
2	5-19	KATLQDIVLHLEPQN
3	9-23	QDIVLHLEPQNEIPV
4	13-27	LHLEPQNEIPVDLLC
5	17-31	PQNEIPVDLLCHEQL
6	21-35	IPVDLLCHEQLSDSE
7	25-39	LLCHEQLSDSEEEND
8	29-43	EQLSDSEEENDEIDG
9	33-47	DSEEENDEIDGVNHQ
10	37-51	ENDEIDGVNHQHLPA
11	41-55	IDGVNHQHLPARRAE
12	45-59	NHQHLPARRAEPQRH
13	49-63	LPARRAEPQRHTMLC
14	53-67	RAEPQRHTMLCMCCK
15	57-71	QRHTMLCMCCKCEAR
16	61-75	MLCMCCKCEARIKLV
17	65-79	CCKCEARIKLVVESS
18	69-83	EARIKLVVESSADDL
19	73-87	KLVVESSADDLRAFQ
20	77-91	ESSADDLRAFQQLFL
21	81-95	DDLRAFQQLFLNTLS
22	85-99	AFQQLFLNTLSFVCP
23	89-103	LFLNTLSFVCPWCAS
24	93-107	TLSFVCPWCASQQ

**Table 2:** **HPV type 18 E7**_**81–95**_ **and E**_**89–103**_ **truncated peptides**

**Number**	**Amio acid position**	**Amino acid sequence**	**Number**	**Amio acid position**	**Amino acid sequence**
21-1	82-95	DLRAFQQLFLNTLS	23-1	90-103	FLNTLSFVCPWCAS
21-2	83-95	LRAFQQLFLNTLS	23-2	91-103	LNTLSFVCPWCAS
21-3	84-95	RAFQQLFLNTLS	23-3	92-103	NTLSFVCPWCAS
21-4	85-95	AFQQLFLNTLS	23-4	93-103	TLSFVCPWCAS
21-5	86-95	FQQLFLNTLS	23-5	94-103	LSFVCPWCAS
21-6	87-95	QQLFLNTLS	23-6	95-103	SFVCPWCAS
21-7	88-98	QLFLNTLS	23-7	96-103	FVCPWCAS
21-8	81-94	DDLRAFQQLFLNTL	23-8	89-102	LFLNTLSFVCPWCA
21-9	81-93	DDLRAFQQLFLNT	23-9	89-101	LFLNTLSFVCPWC
21-10	81-92	DDLRAFQQLFLN	23-10	89-100	LFLNTLSFVCPW
21-11	81-90	DDLRAFQQLFL	23-11	89-99	LFLNTLSFVCP
21-12	81-89	DDLRAFQQLF	23-12	89-98	LFLNTLSFVC
21-13	81-88	DDLRAFQQL	23-13	89-97	LFLNTLSFV
21-14	81-87	DDLRAFQQ	23-14	89-96	LFLNTLSFV

### Generation of autologous dendritic cells and peptide-specific CTLs

Autologous dendritic cells (DCs) were generated as previously described, with minor modifications [25,26]. PBMCs were incubated for 2 h at 37°C using complete RPMI medium containing 10% fetal bovine serum (FBS). Adherent monocytes were resuspended at a concentration of 5 × 10^6^ cells/mL in complete RPMI medium with granulocyte-macrophage colony-stimulating factor (1500 IU/mL; PeproTech, Rocky Hill, NJ, USA) and interleukin (IL)-4 (1200 IU/mL; PeproTech). On days 2, 4, and 6 of culture, fresh cytokines were added. On day 5 of culture, 10 ng/mL of tumor necrosis factor-α (R&D Systems, Minneapolis, MN, USA) was added for DC maturation. After maturation, autologous DCs were pulsed with peptides for at least 6 h. PBMCs were plated at a concentration of 2 × 10^6^ cells per well in a 24-well culture plate (Nunc, Rochester, NY, USA) with 2 mL of complete RPMI medium. PBMCs were sensitized with synthetic HPV18 E7 peptides (10 μg/mL/well), and 1000 IU/mL/well of recombinant human IL-2 (rhIL-2; PeproTech) was added. Additionally, rhIL-2 (1000 IU/mL/well) was added to the culture every other day. For 1-week expansion, peptide-pulsed autologous DCs (4–10 × 10^6^/well) were added to the PBMCs on day 7, incubated for 6 h, and analyzed with flow cytometry. DC-treated PBMCs were cultured for another 7 days, and cytotoxicity assays were performed.

### Intracellular flow cytometric analysis

Phycoerythrin (PE)-conjugated anti–interferon-gamma (anti-IFN-γ), APC-Cy7-conjugated anti-CD44, FITC-conjugated anti-CD3, PerCp-Cy5.5-conjugated anti-CD4, and APC-conjugated anti-CD8 were purchased from BD Biosciences (San Jose, CA, USA). For each sample, 1 × 10^5^ events were gated on a LSR II flow cytometer (BD Biosciences, Franklin Lakes, NJ, USA). For data analysis (Flowjo software; Tree Star, Ashland, OR, USA), positive cells were expressed as a percentage of the respective reference population. The assessment of responses was previously described in more detail [24]. Briefly, peptide-sensitized PBMCs (1 × 10^6^ cells/mL) stimulated with phytohemagglutinin (PHA, Sigma) and PBMCs pulsed with autologous DCs that were not loaded with any peptide were used as positive and negative controls, respectively. One hour after stimulation, 10 μg of Brefeldin A (Sigma) was added to each well. After 5 additional hours of incubation, PBMCs were washed once with phosphate-buffered saline (PBS) and then incubated in PBS containing 1 mM ethylene-diamine-tetraacetic acid for 10 min. After 2 additional washes with PBS containing 5% FBS, the cells were incubated with fluorescently-labeled monoclonal anti-CD3, anti-CD8, anti-CD4, anti-CD44, and anti–INF-γ antibodies for 15 min on ice in the dark prior to analysis.

### IFN-γ enzyme-linked immunosorbent spot assay (ELISpot)

IFN-γ production was determined in PBMCs stimulated with HPV18 E7 peptides. Cryopreserved PBMCs were thawed into complete RPMI (RPMI supplemented with L-Glutamine, penicillin, streptomycin, and 10% heat inactivated fetal calf serum; Invitrogen). Then, 5 × 10^5^ cells/well were plated in triplicate for each treatment in the ELISpot plates (Millipore, Billerica, MA, USA). Plates were previously coated with anti-IFN-γ antibody (Clone AN-18, Ebioscience, San Diego, CA, USA) at 4°C, overnight. PBMCs were incubated with HPV18 E7 peptides for 48 h at 37°C and 5% CO2. Phytohemagglutinin (PHA) was added at 2.5 μg/mL as a positive control and PBMCs sensitized with no peptide were used as a negative control. Biotinylated IFN-γ detection antibody (100 μL; 1 μg/mL in PBS) was added to each well (clone R4-6A2, Ebioscience, San Diego, CA, USA) and incubated at RT for 1.5 hours. After washing the plate 5 times with PBS, 100 μl of streptavidin-alkaline phosphatase (Invitrogen) diluted 1:1000 in PBS was added. The plate was incubated at RT for 1 h in the dark and developed with color solution by mixing 10 mL of Tris-MgCl_2_ buffer with 100 μL of NBT solution (Bio-Rad, Berkeley, CA, USA) and 100 μL of BCIP solution (Bio-Rad). The plate was dried before reading. The spots were quantified with an ELISpot plate reader and software version 3.5 (AID ELISpot Reader System, Strassberg, Germany). Data were obtained by calculating the means of triplicate wells. ELISpot data were expressed as the total IFN-γ spot-forming units (SFU)/10^6^ PBMCs.

### Cell culture

The human HPV 18+, cervical cancer cell lines (HTB-34 for HLA-A*02:01, SNU-1160-A*2402) were purchased from by the Korean Cell Line Bank (Seoul, Korea), and it has been tested and authenticated. Cancer cell line was expanded and frozen in aliquots within 4 weeks of purchase. Cancer cells were thawed and cultured at 37°C and 5% CO2 in Dulbecco’s modified Eagle medium (Gibco, Grand Island, NY, USA) containing 10% FBS and 1% antibiotics for no more than 8 passages.

Immortalized Epstein-Barr virus-B lymphoblastoid cell lines (EBV-BLC) which were restricted each HLA class I (kindly gifted from Dr. David Stroncek, in NIH) were cultured at 37°C and 5% CO2 in RPMI-1640 with 10% FBS. Cells were routinely tested for the absence of mycoplasma.

### *In vitro* cytotoxicity assay

Cytotoxicity assays were performed using the ^51^Cr release assay. Briefly, cervical cancer cells labeled for 45 min with ^51^Cr (100 mCi/10^6^ cells; Perkin Elmer, Waltham, MA, USA), washed in PBS, and dispensed in triplicate into 96-well U-bottom plates (Nunc, Rochester, NY, USA) at 4 × 10^3^ cells/well. Peptide-sensitized PBMCs were added at an effector: target ratio of either 10:1, 30:1, 50:1, or 100:1. The cells were pelleted and incubated for 6 h, and the supernatant was analyzed using a WIZARD2 Automatic Gamma Counter (Perkin Elmer). Spontaneous and total release for each target were used to calculate the percentage of specific release according to the following formula: % specific release = (experimental counts per minute – spontaneous counts per minute)/(total counts per minute – spontaneous counts per minute) × 100.

### Statistical analysis

Data presented as mean ± standard error are the representative of at least 3 different experiments. To compare between control group and each tested group, a student *t-*test (two-tailed) was used. *P*-values less than 0.05 was considered statistically significant.

## Results

### Screening of 15-amino acid peptides from HPV 18 E7 for elicit of Th1 response

**Figure 1:**
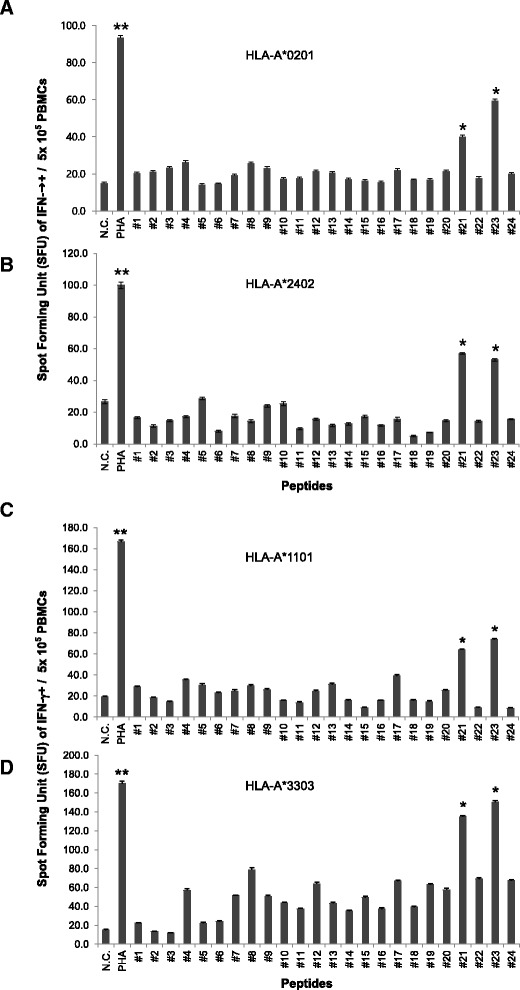
**Screening of immunogenic epitopes of HPV18 E7 which could sensitize PBMCs of four major HLA class I using IFN-γ ELISpot assay.** ELISpot assays were performed to measure IFN-γ production from donor’s PBMCs of four major HLA class I that were sensitized with 24 candidate peptides. HPV18 E7_81–95_ (#21) and E7_89–103_ (#23) induced greater number of IFN-γ^+^ spots from PBMCs of HLA-A*02:01 **(A)**, HLA-A*24:02 **(B)**, HLA-A*11:01 **(C)**, HLA-A*33:03 **(D)** than other peptides. Data are representative of at least three independent experiments using PBMCs from HLA-A*02:01, HLA-A*24:02, HLA-A*11:01, HLA-A*33:03 subjects. PBMCs sensitized with PHA were used as a positive control (PHA), and PBMCs with sensitized with no peptide (No peptide) were used as the negative control (N.C). Data are presented as mean ± standard error. Statistically significant differences between the tested group and negative control group were determined using a student *t*-test (two-tailed). **P < .001; *P < .05.

IFN-γ^+^ spot forming unit (SFU) were counted using ELISpot assay after *in vitro* sensitization of PBMCs with each candidate peptide to determine which 15-amino acid peptides, from the 24 candidate peptides, were able to elicit CTL-specific immune responses. In HLA-A*02:01, A*11:01 and A*33:03, HPV 18 E7_89-103_LFLNTLSFVCPWCAS (#23) and HPV 18 E7_81-95_DDLRAFQQLFLNTLS (#21) consistently induced the highest and 2nd highest production of IFN-γ^+^ spots from PBMCs among 24 candidate peptides, respectively (Figure 1A, C, D). In HLA-A*2402, E7_81–95_ (#21) induced the highest production of IFN-γ + spots and E7_89–103_ (#23) was 2nd highest production of IFN-γ + spots from PBMCs among 24 candidate peptides (Figure 1B). E7_89–103_ (#23) and E7_81–95_ (#21) induced at least 3 fold higher numbers of IFN-γ + spot forming units (SFU) from PBMCs than those of negative control (PBMCs sensitized with no peptide) in all four HLA class I (P < 0.05, P < 0.05, respectively). These results indicated that HPV18 E7_81–95_ (#21) and E7_89–103_ (#23) could induce strong Th1 response from donor PBMCs of HLA-A*02:01, A*24:02, A*11:01, A*33:03 simultaneously. Because Th1 response was mainly induce by CD8^+^ and CD4^+^ T cells as well as NK cells and the CD8^+^ CTLs play a major role in anti-viral and anti-tumor responses, we further investigated to determine whether these two candidate peptides induce CD8+ CTL response using flow cytometry analysis.

### Measuring CD8+ CTL response after sensitization with 15-amino acid peptides

**Figure 2:**
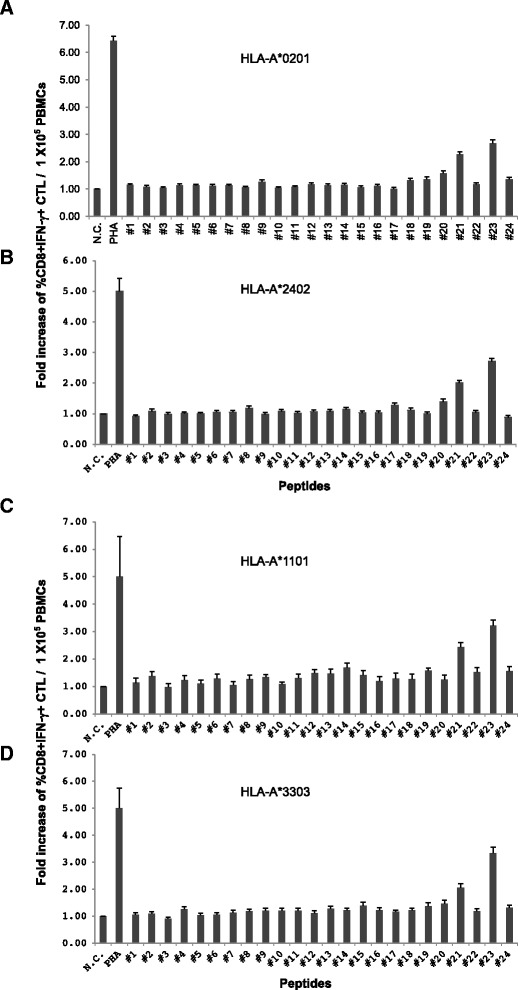
**Fold increase of the percentage of CD8 + IFN-γ + within donor’s PBMCs of four major HLA class I after sensitization of candidate peptides.** The percentage of CD8^+^IFN-γ^+^ from donor’s PBMCs of four major HLA class I were measured to determine whether the CD8^+^ CTL response were induced by sensitization of donor PBMCs with candidate peptides and the fold increase of the percentage of CD8^+^IFN-γ^+^ within the total CD8^+^ T cell population in donor PBMCs after re-sensitization of each candidate peptide were compared to that of negative control (PBMCs sensitized with no peptide). HPV18 E7_81-95_DDLRAFQQLFLNTLS (#21) and E7_89-103_LFLNTLSFVCPWCAS (#23) consistently induced higher the percentage of CD8^+^IFN-γ^+^ T cells than that of other candidate peptides in HLA-A*02:01 **(A)**, HLA-A*24:02 **(B)**, HLA-A*11:01 **(C)**, HLA-A*33:03 **(D)**. PBMCs sensitized with PHA were used as a positive control (PHA), and PBMCs sensitized with no peptide (No peptide) were used as the negative control (N.C). Data are presented as mean ± standard error.

We measured the intracellular IFN-γ production from four HLA class I (HLA-A*02:01, A*24:02, A*11:01, A*33:03) donor’s PBMCs to determine if the CD8^+^ CTL response could be induced by sensitization of donor PBMCs with candidate peptides. After a week of *in vitro* sensitization, PBMCs were restimulated with dendritic cells derived from autologous monocytes that were loaded with each candidate peptide. After a 6-hour resensitization, intracellular IFN-γ production from donor’s CD8^+^ T cells (CD3^+^CD8^+^IFN-γ^+^) was measured by flow cytometry. The fold increases of the percentage of CD8^+^ T cells that produced intracellular IFN-γ (CD3^+^CD8^+^IFN-γ^+^) after resensitization of candidate peptides among the total CD3^+^CD8^+^ T cell population were calculated and compared to that of the negative control (CD3^+^CD8^+^IFN-γ + among PBMCs sensitized with no peptide) (Figure 2). HPV18 E7_81–95_ (#21) and E7_89–103_ (#23) consistently induced higher percentage of CD3^+^CD8^+^IFN-γ^+^ than that of other candidate peptides in HLA-A*02:01, A*24:02, A*11:01, A*33:03. E7_81–95_ (#21) and E7_89–103_ (#23) showed at least 2-fold and 2.5-fold higher induction of CD8^+^IFN-γ^+^ T cells compared to the negative control in HLA-A*02:01, A*24:02, A*11:01, A*33:03, respectively.

These results suggested that E7_81–95_ (#21) and E7_89–103_ (#23) could successfully induce CD8+ CTL response from PBMC and these two peptides were selected for further study.

### Analysis of the peptide-specific cytotoxicity of E7_81–95_ (#21) and E7_89–103_ (#23) peptides

**Figure 3:**
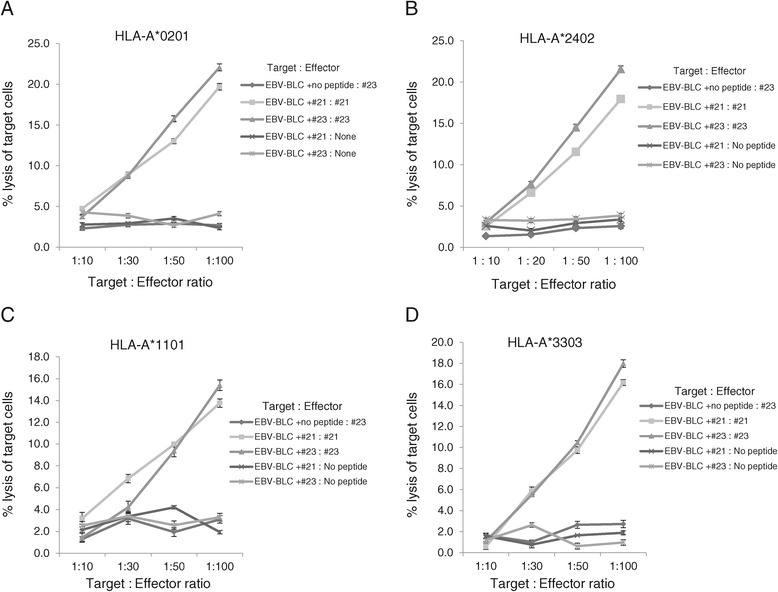
**EBV-BCLs cytotoxicity of PBMCs sensitized with HPV 18 E7**_**81-95**_ **(#21) and E7**_**89-103**_ **(#23) peptides.** HPV18 E7_81-95_ (#21)- and E7_89-103_ (#23)-sensitized CTLs lysed more EBV-BLCs with E7_81-95_ (#21) and E7_89-103_ (#23), respectively, than negative control (EBV-BLCs with no peptide) in all HLA-A*02:01 **(A)**, A*24:02 **(B)**, A*11:01**(C)**, A*33:03 **(D)**. PBMCs sensitized with E7_81-95_ or E7_89-105_ with EBV-BCLs loaded with no peptide or PBMCs sensitized with no peptide and E7_89-105_-loaded EBV-BCLs were used as negative controls. Data are representative of at least three independent experiments using PBMCs from HLA-A*02:01, HLA-A*24:02, HLA-A*11:01, HLA-A*33:03 subjects. Data are presented as mean (point) ± standard error (bar).

To confirm that HPV18 E7_81–95_ (#21) and E7_89–103_ (#23) are immune-dominant peptides for each HLA class I subjects, PBMCs from four HLA class I donors (HLA-A*02:01, A*24:02, A*11:01, A*33:03) were sensitized *in vitro* for two weeks with the candidate peptides and were tested for cytotoxic effects using the HLA-matched EBV-BLCs as the target cells because EBV-BLCs could present each peptide successfully in the HLA-peptide complex on their cell surface. HPV18 E7_81–95_ (#21)- and E7_89–103_ (#23)-sensitized CTLs lysed more EBV-BLCs loaded with E7_81–95_ (#21) and E7_89–103_ (#23, respectively, than negative control (EBV-BLCs loaded with no peptide) in all HLA-A*02:01, A*24:02, A*11:01, A*33:03 (Figure 3A-D). PBMCs sensitized for 2 weeks with E7_89–103_ (#23) were slightly higher cytotoxicity to HLA-matched EBV-BLCs loaded with E7_89–103_ (#23) than that of E7_81–95_ (#21) in all four HLA class I. These results confirmed that both HPV-18 E7_81–95_ (#21) and E7_89–103_ (#23) were successfully recognized by CD8+ CTLs from 4 different HLA-A and induced CD8+ cytotoxicity response in all four HLA class I (HLA-A*02:01, A*24:02, A*11:01, A*33:03).

### Cervical cancer cell cytotoxicity of PBMCs sensitized with E7_81–95_ (#21) and E7_89–103_ (#23) peptides

**Figure 4:**
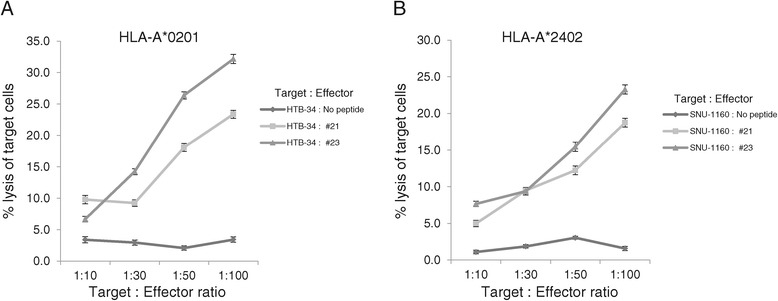
**Cervical cancer cell cytotoxicity of PBMCs sensitized with HPV 18 E7**_**81-95**_ **(#21) and E7**_**89-103**_ **(#23) peptides.** Cytotoxicity assays with HLA-matched cervix cancer cell lines were tested using the ^51^Cr-release assay to confirmed HPV18 E7_81-95_ (#21)- and E7_89-103_ (#23)-sensitized PBMCs could successfully recognized same peptides that naturally presented on the surface of cancer cells. E7_81-95_ (#21)- and E7_89-103_ (#23)-sensitized PBMCs of HLA-A*02:01 and HLA-A*24:02 lysed greater quantities of HTB-34 cells (HPV 18+, HLA-A*02:01) **(A)** and SNU-1160 (HPV 18+, HLA-A*24:02) **(B)** than that of negative control (PBMCs sensitized with no peptide), respectively. Data are representative of at least three independent experiments using PBMCs from HLA-A*02:01, HLA-A*24:02, HLA-A*11:01, HLA-A*33:03 subjects. Data are presented as mean (point) ± standard error (bar).

To confirm the cervical cancer cell cytotoxicity of PBMCs sensitized E7_81–95_ (#21) and E7_89–103_ (#23) peptides, PBMCs from four HLA-A*02:01, A*24:02 donors were sensitized with E7_81–95_ (#21) and E7_89–103_ (#23) peptides *in vitro* for two weeks and were tested for cytotoxic effects on the HLA-matched cervical cancer cell line (HTB-34 restricted to HLA-A*02:01 and SNU-1160 restricted to HLA-A*24:02). The cytotoxicity assay was carried out by measuring ^51^Cr release from each HLA-restricted cancer cells. HPV18 E7_81–95_ (#21)- and E7_89–103_ (#23)-sensitized PBMCs lysed more cervical cancer cells than the unsensitized cells. PBMCs from HLA-A*02:01 donors that were *in vitro-*sensitized for 2 weeks with HPV18 E7_89–103_ (#23) and E7_81–95_ (#21) were highly cytotoxic to HLA-A*02:01-restricted HTB-34 cell lines (Figure 4A). PBMCs from HLA-A*24:02 donors that were *in vitro-*sensitized for 2 weeks with HPV18 E7_89–103_ (#23) and E7_81–95_ (#21) were also highly cytotoxic to SNU-1160 cells (Figure 3B). These results confirmed that both HPV-18 E7_81–95_ (#21) and E7_89–103_ (#23) epitopes were naturally processed in both HTB-34 and SNU-1160 cervical cancer cell lines and were to be useful as the HLA-A*02:01 and HLA-A*24:02 immunogenic epitopes within the HPV18 E7 protein.

### Determination of the specific residues within E7_81–95_ (#21) and E7_89–103_ (#23) epitopes recognized by CD8^+^ CTLs

**Figure 5:**
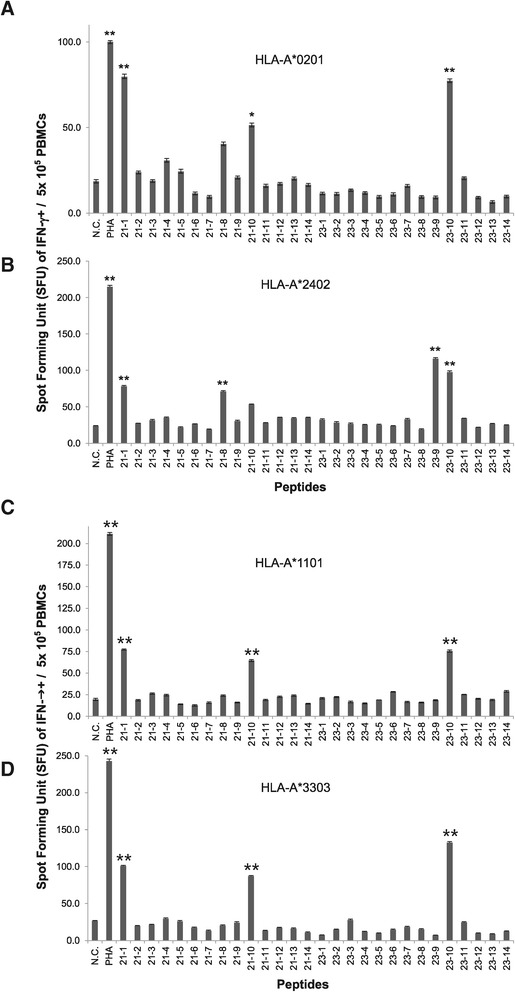
**Determination the fine specific residues within the 15-aa epitopes recognized by PBMCs using IFN-γ ELISpot assay.** IFN-γ ELISpot assays were performed to determination the fine specific residues within HPV18 E7_81–95_(#21) and E7_89–103_ (#23) which were recognized by PBMCs. In HLA-A*02:01 **(A)**, residue 82 of the N-terminus and 92, 94 of C-terminus within HPV18 E7_81–95_ (#21) were important to elicit Th1 response of PBMCS. Residue 100, 103 of C-terminus within E7_89–103_ (#23) were important to elicit the cytotoxic response of PBMCs. In HLA-24:02 **(B)**, residue 82 of the N-terminus and 92, 94 of C-terminus within HPV18 E781-95 (#21) were important to elicit Th1 response of PBMCS. Residue 100, 101, 103 of C-terminus within E789-103 (#23) were important to elicit the cytotoxic response of PBMCs. In HLA-A*11:01 **(C)** and 33:03 **(D)**, residue 82 of the N-terminus and 94 of C-terminus within HPV18 E7_81–95_ (#21) were important to elicit Th1 response of PBMCS. Residue 100, 103 of C-terminus within E7_89–103_ (#23) were important to elicit the cytotoxic response of PBMCs. Data are representative of at least three independent experiments using PBMCs from HLA-A*02:01, HLA-A*24:02, HLA-A*11:01, HLA-A*33:03 subjects. Data are presented as mean ± standard error. Statistically significant differences between the tested group and negative control group were determined using a student *t*-test (two-tailed). **P < .001; *P < .05.

To determine the specific residues within E7_81–95_ (#21) and E7_89–103_ (#23) epitopes recognized by CD8^+^ CTLs, a panel of N- and C-terminal truncations of HPV18 E7_81–95 _(#21) and E7_89–103_ (#23) was synthesized (Table 2) and tested using ELISpot assay (Figure 5). In all four HLA types, the stepwise removal of amino acids from the N-terminal end of the HPV-18 E7_81–95_ (#21) revealed that the Th1 response was diminished when residue 82 was deleted. This result suggested that residue 81 and 82 of the N-terminus were important to elicit Th1 response of PBMCs in four different HLA class I (Figure 5A-D). In case of HLA-A*02:01 and HLA-24:02, truncations from the C-terminal side of HPV-18 E7_81–95_ (#21) revealed that residue 92, 94, and 95 were important for the CD8^+^ CTL response. These results indicate that residues 92, 94 and 95 would mark the C-terminal edge of the CD8^+^ CTL-stimulating epitope (Figure 5A and B). However, in case of HLA-A*11:01 and HLA-33:03, residue 94 and 95 were important for the PBMCs response, and these residues would mark the C-terminal edge of the CD8^+^ CTL-stimulating epitope (5C, 5D).

In HLA-A*02:01, A*11:01 and A*33:03, truncations from the C-terminal end of HPV18 E7_89–103_ (#23) revealed that residue 100 and 103 were important to elicit the CD8^+^ CTL response. This suggested that residues 100 and 103 comprise the C-terminal end of the CD8^+^ CTL-stimulating epitope (5A, 5C, 5D). However, in case of HLA-A*24:02, residue 100, 101 and 103 were important to elicit the CD8^+^ CTL response and residues 100, 101 and 103 comprise the C-terminal end of the CD8^+^ CTL-stimulating epitope (5B).

### Truncated peptides of HPV-18 E7_81–95_ (#21) and E7_84–99_ (#23) induced the peptide-specific cytotoxicity

**Figure 6:**
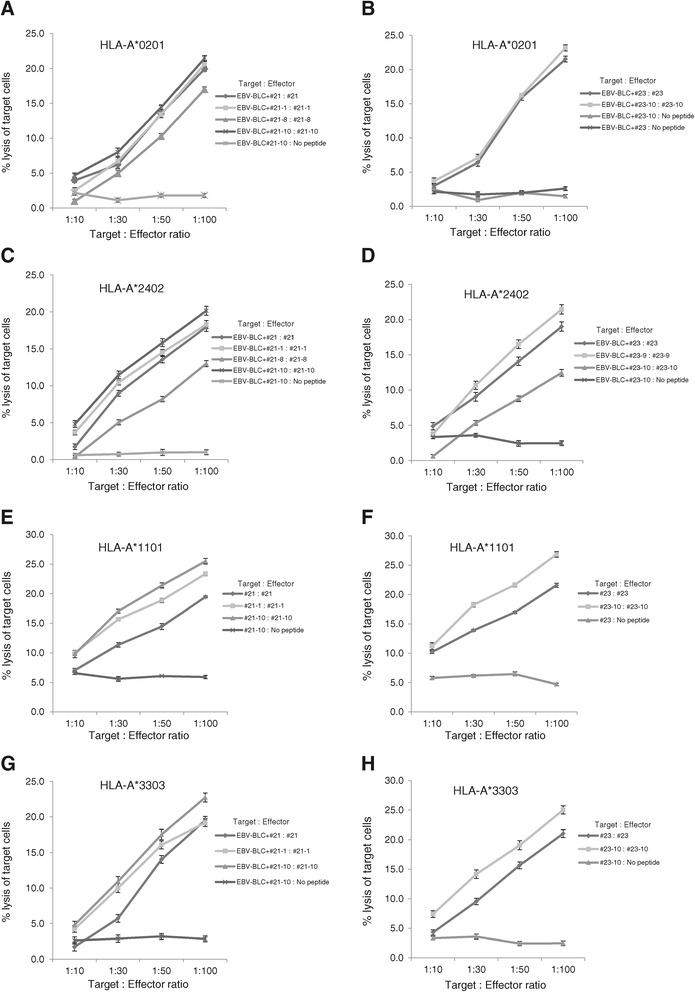
**Truncated peptides of HPV-18 E7**_**81–95**_ **(#21) and E7**_**84–99**_ **(#23) induced the peptide-specific cytotoxicity.** PBMCs were sensitized with truncated peptides and tested for cytotoxicity using the ^51^Cr release assay against HLA-matched, truncated peptide-loaded EBV-BLCs. In HLA-A*02:01, truncated peptides #21-1, #21-8, #21-10 and #23-10-sensitized CTLs lysed more EBV-BLCs loaded with same peptide, respectively, than negative control **(A,B)**, In HLA-A*24:02, #21-1, #21-8, #21-10 and #23-9, #23-10-sensitized CTLs lysed more EBV-BLCs loaded with same peptide, respectively, than negative control **(C,D)**. In HLA-A*11:01, #21-1, #21-10 and #23-10-sensitized CTLs lysed more EBV-BLCs loaded with same peptide, respectively, than negative control **(E,F)**. In HLA-A*33:03, #21-1, #21-10 and #23-10-sensitized CTLs lysed more EBV-BLCs loaded with same peptide, respectively, than negative control **(G,H)**. PBMCs sensitized with truncated-peptide and EBV-BCLs loaded with no peptide were used as the negative control. Data are representative of at least three independent experiments using PBMCs from HLA-A*02:01, HLA-A*24:02, HLA-A*11:01, HLA-A*33:03 subjects. Data are presented as mean (point) ± standard error (bar).

To provide further evidence that truncated peptides of HPV18 E7_81–95_ (#21) and E7_84–99_ (#23) induced epitope-specific and HLA class I-restricted cell cytotoxicity, PBMCs were sensitized with the truncated peptides and tested for cytotoxicity, using the ^51^Cr release assay with HLA-matched EBV-BLCs loaded with same peptides. In case of HLA-A*02:01 and A*24:02, HPV18 E7_82-95_DLRAFQQLFLNTLS (#21-1), E7_81-94_DDLRAFQQLFLNTL (#21-8), and E7_81–92_ DDLRAFQQLFLN (#21-10) from HPV18 E7_81–95_ (#21)-sensitized CTLs lysed greater quantities of EBV-BLCs than that of negative control cells (EBV-BLCs loaded with no peptide or PBMCs sensitized with no peptide) (Figure 6A and C). In HLA-A*11:01 and A*33:03, HPV18 E7_82-95_DLRAFQQLFLNTLS (21–1) and E7_81–92_ DDLRAFQQLFLN (21–10) from HPV18 E7_81–95_ (#21)-sensitized CTLs lysed greater quantities of EBV-BLCs than that of negative control cells (EBV-BLCs pulsed with no peptide (Figure 6E and G). In case of HLA-A*02:01, A*11:01 and A*33:03, CTLs sensitized with E7_84–97_ LFLNTLSFVCPW (#23-10) from HPV18 E7_89–103_ (#23) lysed greater quantities of EBV-BLCs loaded with same peptide than the negative controls (EBV-BLCs loaded with no peptide or PBMCs sensitized with no peptide)) (Figure 6B, F, H). In HLA-A*24:02, CTLs sensitized with E7_84–98_ LFLNTLSFVCPWC (23–9) and E7_84–97_ LFLNTLSFVCPW (23–10) from HPV18 E7_89–103_ (#23) lysed greater quantities of EBV-BLCs pulsed with same peptide than the negative controls (EBV-BLCs loaded with no peptide) (Figure 6D). These results were comparable to the results of ELISpot assays in Figure 5.

## Discussion

A strong Th1-biased T-cell response is important for control or elimination of not only infection of HPV 16 and 18 in precancerous disease, but also HPV-induced cervical cancer. E6 and E7 protein of HPV have been suggested as good targets for immune monitoring or immunotherapy against HPV-associated disease because E6 and E7 are constitutively expressed in both HPV-infected cells and cervical cancer cells and are not express in normal cervical epithelia. Therefore, the identification of CTL specific epitopes from E6 and E7 proteins of HPV 16 and 18 is essential for the development of peptide-based immune monitoring of HPV-specific CTL response and immunotherapy in patients. However, a major drawback of the development of clinically effective peptide-base immune monitoring or immunotherapy is the fact that different epitopes are recognized by T cells from individuals displaying distinct major HLA molecules. Moreover, although HPV 18 is second most common subtype after HPV 16 which is associated with cervical cancer, only few CTL-specific epitopes of HPV18 have been identified.

Until very recently, the studies searching for immune dominant peptides were performed testing of substantial numbers of overlapping peptides or peptide libraries. The identification of major HLA-binding motifs allowed the prediction of potential T cell epitopes (21,22), and supertypes or single peptide which can sensitize multiple HLA molecules simultaneously were found (23). However, very few studies have addressed CTL recognition of single peptide in a genetically heterogeneous group previously exposed to HPV infection or cervical cancer.

In this study, we found that two overlapping 15-amino acid peptides from HPV 18 E7 protein, E7_81-95_DDLRAFQQLFLNTLS (#21) and E7_89-103_LFLNTLSFVCPWCAS (#23), which could sensitize PBMCs of four major HLA class I A molecules including HLA-A*02:01, A*24:02, A*1101, and A*33:03, simultaneously. These four major HLA class I alleles are the most frequent HLA class I allele in human race. These 15-amino acid peptides were also successfully recognized by CD8+ CTLs of four different HLA class I alleles.

In the screening test, E7_81–95_ (#21) and E7_89–103_ (#23) were selected because these peptides induced most strong IFN-γ responses from PBMCs of four different HLA class I than other peptides (Figure 1A-D). However, IFN-γ could be released from many kinds of immune cells, including CD8^+^ and CD4^+^ T cells and NK cells. Thus, to determine if IFN-γ was produced from CD8^+^ T lymphocytes, CD3^+^CD8^+^IFN-γ^+^ T cells were counted using flow cytometry, and HPV-18 E7_81–95_ (#21) and E7_89–103_ (#23) induced greater numbers of CD3^+^CD8^+^IFN-γ^+^ T cells than other peptides and negative control (PBMCs sensitized with no peptide) in all four different HLA types (Figure 2A-D). HPV18 E7_81–95_ (#21)- and E7_89–103_ (#23)-sensitized CTLs lysed more EBV-BLCs loaded with E7_81–95_ (#21) and E7_89–103_ (#23), respectively, than negative control (EBV-BLCs with no peptide) in all four HLA types (Figure 3A-D). It suggested that these epitopes successfully bound to HLA-A*02:01, 24:02, 11:01 and 33:03 molecules on the surface of EBV-BLCs and also were successfully recognized by CD8^+^ CTLs within the PBMCs that were in vitro sensitized with same peptides. HPV18 E7_81–95_ (#21)- and E7_89–103_ (#23)-sensitized HLA-A*02:01 and 24:02 PBMCs also lysed more HLA-matched cervical cancer cell lines (HTB-34 and SNU-1160, respectively) than negative control (PBMCs sensitized with no peptide) (Figure 5A, B). These results confirmed that both HPV-18 E7_81–95_ (#21) and E7_89–103_ (#23) epitopes were naturally processed in both HTB-34 cell lines and SNU-1160 cell lines and were to be useful HLA-A*02:01 and HLA-A*24:02 immunogenic epitopes within the HPV18 E7 protein. We only used HTB-34 and SNU-1160 for cytotoxicity assay against cervical cancer cell in this study because these two cervical cancer cell lines were only available HPV 18+ E7 expressed cell lines in ATCC and Korean Cell Line Bank.

Virally infected human cells or human cancer cells can be recognized by CD8^+^ CTLs through immunogenic epitopes of 8 to 12 amino acids that are presented on the cell surface with HLA class I molecules. Thus, we used truncated peptides to determine the critical residues that induce CD8^+^ CTL responses within the 15-amino acid peptides and to determine the position of MHC anchor residues. Residue 81,82 of the N-terminus of E7_81–95_ (#21) were important to elicit Th1 response of PBMCS in all four HLA class I (Figure 5A-D). Residue 92, 94, 95 would mark the C-terminal edge of the CD8^+^ CTL-stimulating epitope in HLA-A*02:01 and 24:02 (Figure 5A, B). In HLA-A*11:01 and 33:03, residue 94, 95 would mark the C-terminal edge of the CD8^+^ CTL-stimulating epitope within E7_81–95_ (#21) (5C, 5D). In HLA-A*02:01, A*11:01 and A*33:03, residue 100 and 103 comprise the C-terminal end of the CD8^+^ CTL-stimulating epitope in peptide #23 (5A, 5C, 5D). However, in case of HLA-A*24:02, residue 100, 101 and 103 comprise the C-terminal end of the CD8^+^ CTL-stimulating epitope within E7_89–103_ (#23) (5B). These results were comparable to the results of cytotoxicity assays using PBMCs sensitized with truncated peptides within E7_81–95_ (#21) and E7_89–103_ (#23), and HLA-matched EBV-BLCs loaded with same truncated peptides in Figure 6.

In conclusion, we identified E7_81-95_DDLRAFQQLFLNTLS (#21) and E7_89-103_LFLNTLSFVCPWCAS (#23), which could sensitize PBMCs of four HLA class I A molecules including HLA-A*02:01, A*24:02, A*11:01, and A*33:03, simultaneously, and demonstrated that PBMCs sensitized with these peptides showed cytotoxicity against cervical cancer cells. These epitopes could be useful for immune monitoring or immunotherapy against for HPV18-related diseases including cervical cancer, anal cancer, and oropharyngeal cancer.
